# Ruth Beutler: the woman behind Karl von Frisch

**DOI:** 10.1007/s00359-023-01622-0

**Published:** 2023-05-09

**Authors:** Günther K. H. Zupanc

**Affiliations:** Laboratory of Neurobiology, Department of Biology, Northeastern University, Boston, MA 02115 USA

**Keywords:** Comparative physiology, Dance communication, Honeybees, Karl von Frisch, Ruth Beutler, Zeitschrift für vergleichende Physiologie

## Abstract

The Journal of Comparative Physiology A was founded in 1924 as the *Zeitschrift für vergleichende Physiologie* by Karl von Frisch and Alfred Kühn. Given the marginalization of women in science at that time, it is remarkable that the first article in the Journal was authored by a female scientist, Ruth Beutler. Throughout her scientific career, she was affiliated with the Zoological Institute of the University of Munich, which, under the leadership of von Frisch, evolved into a world-class academic institution. Despite chronic health problems, Beutler was one of the first women who succeeded in obtaining the *Habilitation* as qualification for appointment to a professorial position. She was also one of the first scientists who applied methods from physiological chemistry to the study of zoological phenomena. Yet, for many years she was employed as a technician only, and she was never appointed to an *Ordinarius* (tenured full professorship) position. Her most important contributions to comparative physiology outside her own area of research were her support for, and protection of, Karl von Frisch, particularly during the Nazi era when he, as a ‘quarter-Jew,’ faced imminent threat of forced retirement; and after World War II, when her efforts as interim *Ordinarius* were instrumental in re-building the bombed-out Zoological Institute to persuade Karl von Frisch to return to Munich. It was also one of her observations that prompted him to revisit, and revise, his earlier (incorrect) model of how honeybees communicate, through their dances, the direction and distances of food sources from the hive.

## Introduction

**Fig. 1:**
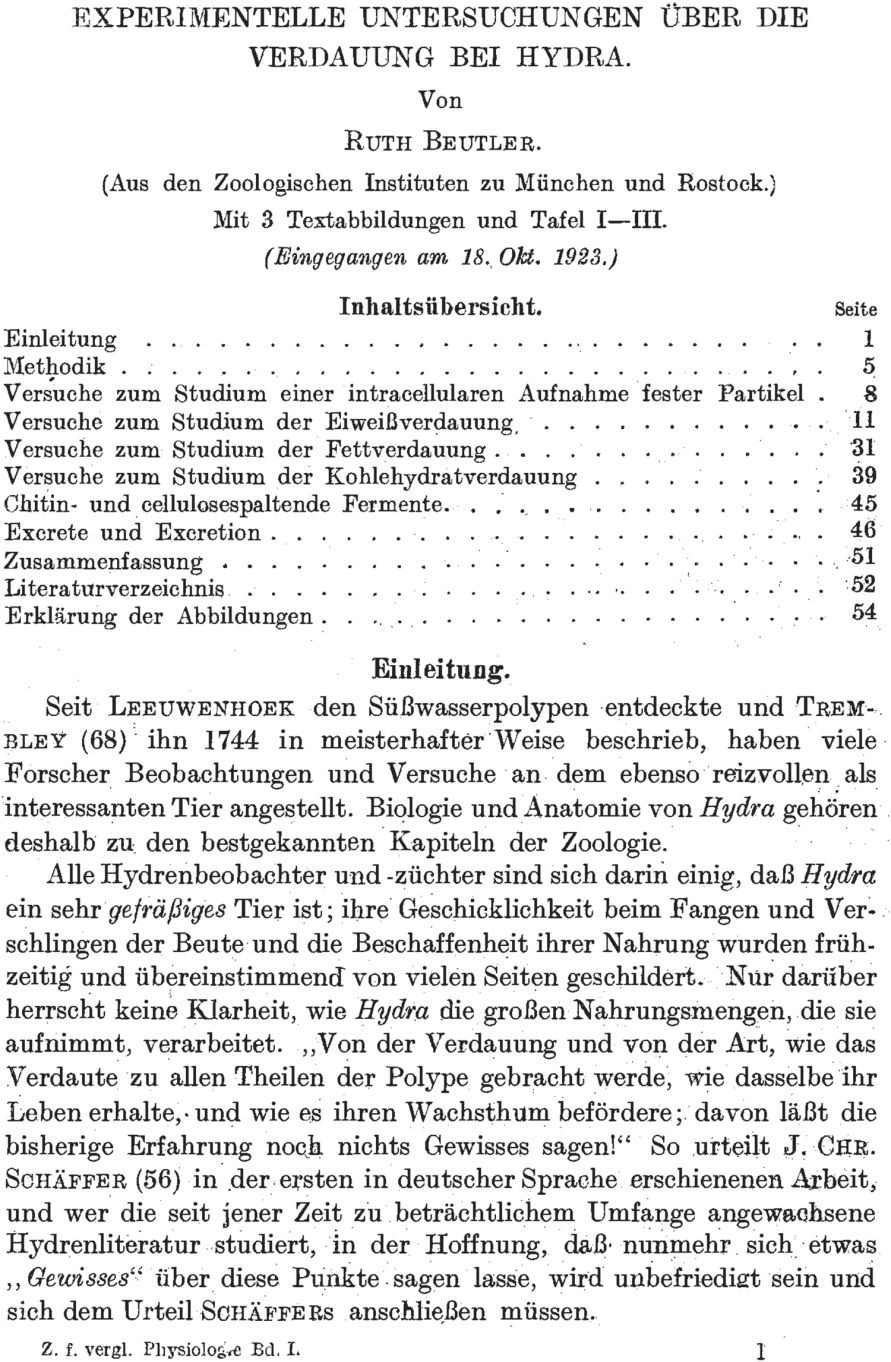
Title page of the first article published in the *Zeitschrift für vergleichende Physiologie*. The paper, authored by Ruth Beutler, is entitled ‘Experimental Studies of Digestion in *Hydra*’. Reproduced from Beutler (1924). With kind permission of Springer-Verlag

The Journal of Comparative Physiology A was founded in 1924 as the *Zeitschrift für vergleichende Physiologie* by Karl Ritter^1^ von Frisch^2^ and Alfred Kühn^3^ (for a historical account see Zupanc 2022). It was the first journal that provided a forum for publication of studies in comparative physiology. Back then, this discipline, pioneered by von Frisch, was still in its infancy. Like science in general (for reviews see Schiebinger 1987; Brush 1991; Kass-Simon et al. 1993; Des Jardins 2010; Watts 2013), comparative physiology was dominated by male scholars. Given this marginalization of females, it is particularly remarkable that the very first article published in the *Zeitschrift für vergleichende Physiologie* (Beutler 1924) (Fig. 1) was authored by a woman, Ruth Beutler. Nevertheless, a century and nearly ten thousand articles later, hardly anyone, including most comparative physiologists, recognizes her name.

I, therefore, set out to research the life and work of Ruth Beutler by retrieving information from the web, including public databases such as PubMed and Google Scholar; archivalia stored in the Bundesarchiv Berlin-Lichterfelde (BAB), the Bayerisches Hauptstaatsarchiv (BayHStA), the Bayerische Staatsbibliothek (BSB), the Staatsarchiv München (StAM), the Stadtarchiv München (StdA), and the Universitätsarchiv München (UAM); as well as by conducting interviews with Sibylle Köhler,^4^ a niece of Ruth Beutler. Translation of German texts into English was done by GKHZ, unless otherwise noted.

What emerged from this work is a picture of a woman who, despite severe health problems that plagued her throughout most of her life, is distinguished by remarkable achievements based on her own research. Perhaps even more significant was her continued, undivided loyalty to, and support of, Karl von Frisch, without which his life and work is likely to have taken a different route.

## Early life

### Family influence

**Fig. 2:**
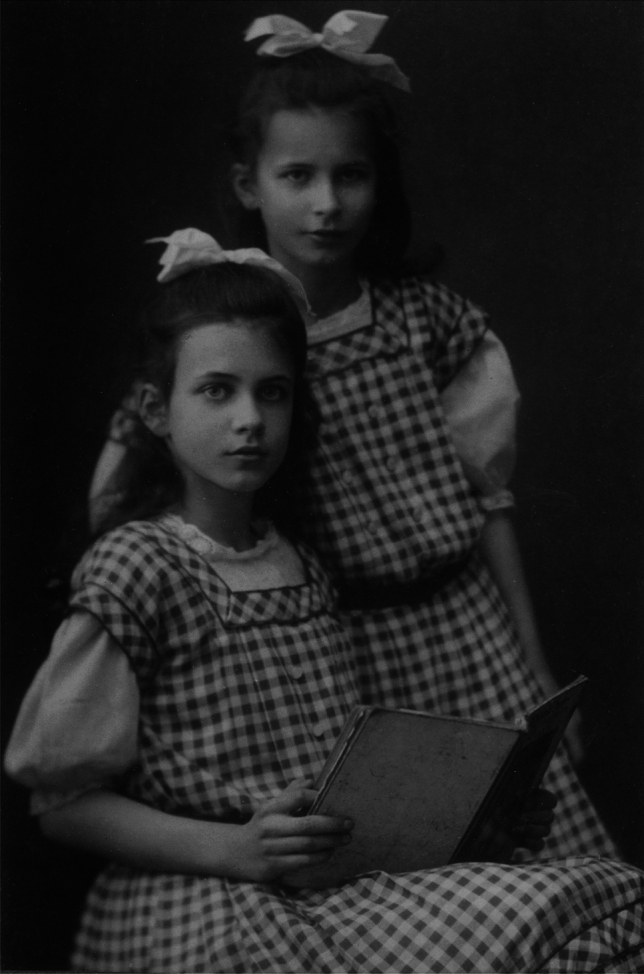
Ruth Beutler (*left*) with her sister Brigitte (*right*). The photograph was taken by Johann Niclou, Photographische Kunst-Anstalt Chemnitz, probably around 1907. Since the copyright term for *Lichtbildwerke* (artistic photographs) in Germany is the author’s lifetime plus 70 years, it is likely that this photograph is in the public domain

Ruth Beutler was born into an influential family in Chemnitz, Germany, on July 16, 1897.^5^ Figure 2 shows her, together with her sister Brigitte, around the age of ten. Her family’s ancestral roots can be traced back to farmers in the sixteenth century. Over the following centuries, many of them served as protestant pastors, including her paternal grandfather.

**Fig. 3:**
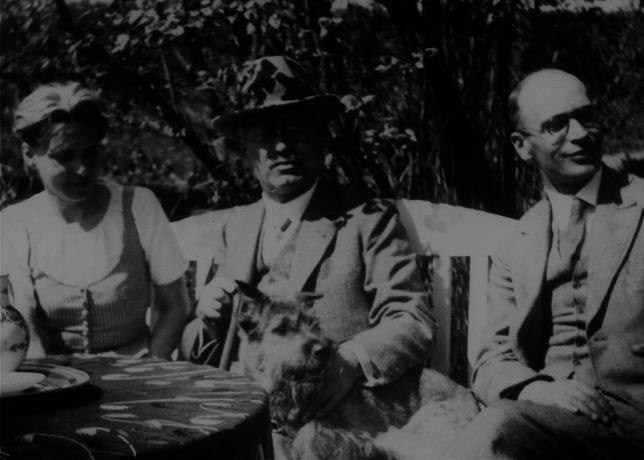
Ruth Beutler with her father Moritz Beutler, her brother Otto Beutler, and the family dog Bazi. Photographer unknown. Since the copyright term for *Lichtbilder*, such as family pictures, in Germany is 50 years after their creation, this photograph is in the public domain

Ruth’s father, Moritz Beutler (1872–1942) (Fig. 3), was a prominent lawyer and notary public in Chemnitz, member of the supervisory boards of numerous German stock corporations, and a powerful national-conservative politician who served as head of the city council and an elected representative for the *Deutschnationale Volkspartei*^6^ in the *Sächsische Volkskammer* (Saxon State Parliament) in the Weimar Republic. In 1911, he bought a manor in Herold (Saxony), the *Rittergut Thum*. Later, Ruth remembered the day when she and her father visited the estate for the first time:

*When I saw the Rittergut Thum, I immediately fell in love … Never the thought crossed my mind that there could be a nicer place anywhere else.*^7^

While the farm was run by estate managers most of the time, the Beutlers spent their weekends and holidays there. Over the years, Moritz Beutler developed the initially rather run-down estate into a modern model farm. In her notes, Ruth Beutler describes how much she had enjoyed gardening and getting involved in farming at the estate. She established her own beehive, and later she took students there for field courses. In 1945, she witnessed the *Rittergut Thum* to burn down when it was hit by incendiary bombs as part of the allied air raid that targeted Dresden and surrounding towns. The family finally lost the estate when they were expropriated by the Soviet occupying authorities in 1946.

Ruth’s brother Otto Beutler (1894–1944) (Fig. 3), like their father, was a conservative national. He served as a career officer in the Imperial German Army, the *Reichswehr*, and the *Wehrmacht* and had risen to the rank of Major General when he was killed in action in Brody (Ukraine). In 1943, during a dinner with two younger officers, he commented on the devastating losses the German Army had suffered during the battle of Stalingrad, and he made derogatory remarks about the German leadership, including Hitler. After Otto Beutler was denounced by one of the officers, he faced trial by a military tribunal during which the prosecution sought the death penalty. He was acquitted only because the other officer committed perjury by denying that this conversation had taken place. However, if Beutler had not been killed in action, he would most likely have faced retrial and been sentenced to death, which Heinrich Himmler^8^ had ordered. The outcome is even more likely, as Beutler was a member of the military resistance that attempted to assassinate Adolf Hitler on July 20, 1944, and to overthrow the Nazi Germany government (Fröhlich 2018).

Ruth’s uncle, Gustav Otto Beutler (1853–1926), was a lawyer in public administration and a conservative politician. He served for 20 years as mayor of Dresden.

Ruth shared with her father his interest in farming and, at least part, of his political views. After graduating from the *Städtische Studienanstalt* in Chemnitz with the *Abitur* (high school diploma) in 1917, she received some practical training in farming and enrolled in an agriculture program at the University of Halle. When the DNVP was founded in 1918, she joined this political party and remained a member until it dissolved itself in 1933.

### Disability and other chronic health problems

**Fig. 4:**
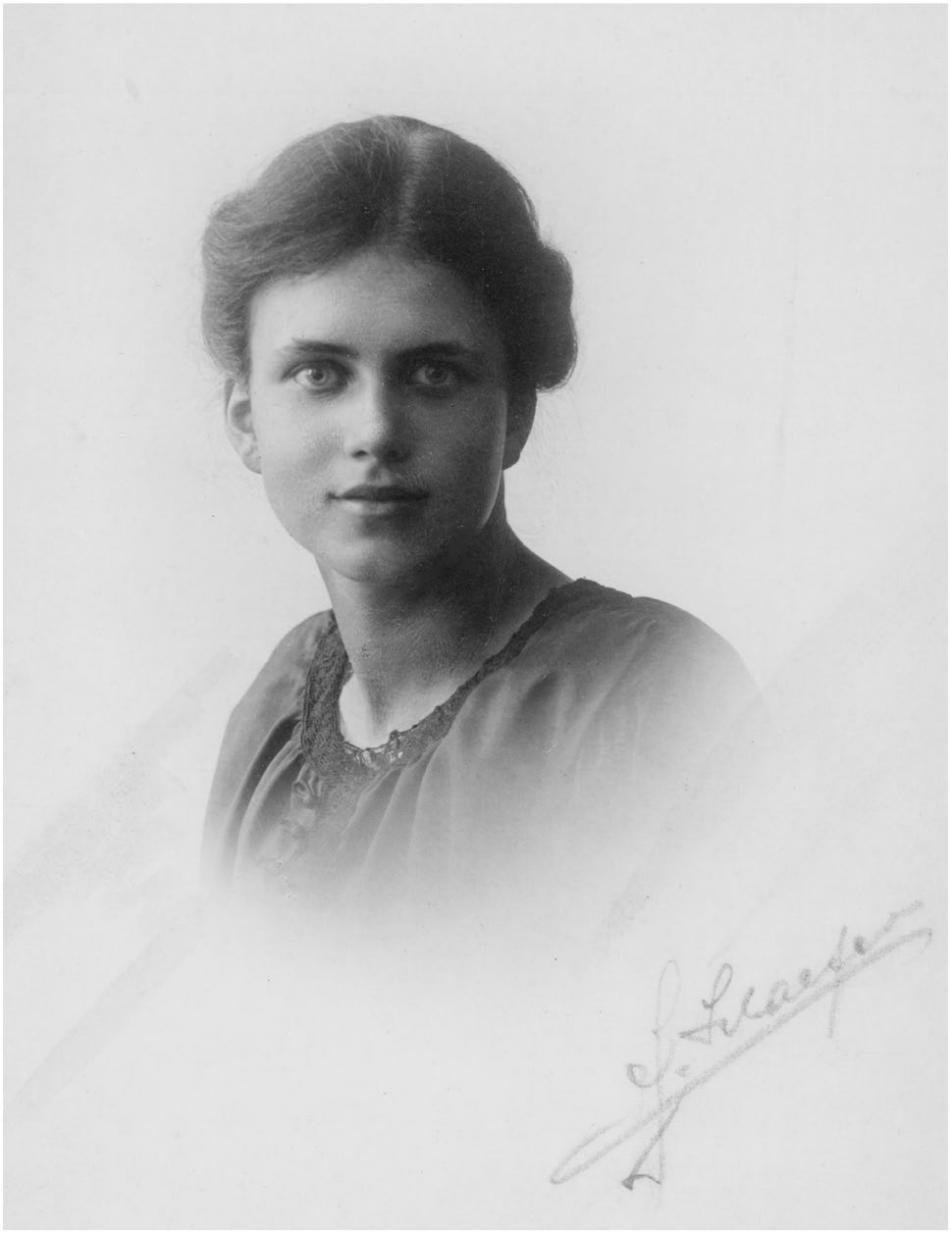
Portrait photograph of Ruth Beutler, probably in her late teens. Photographer unknown. Since the copyright term for *Lichtbildwerke* (artistic photographs) in Germany is the author’s lifetime plus 70 years, it is likely that this photograph is in the public domain

According to Ruth Beutler’s resume,^9^ she was on medical leave from Easter 1918 to Easter 1919. The photograph in Fig. 4 shows her around that time. While her resume does not reveal any details of her illness, an official medical note issued by the public health department of Munich on June 21, 1939, provides possible clues.^10^ It mentions that she wore a prosthetic leg because her right leg had been amputated as a result of osteomyelitis in her adolescent years, without specifying the exact time of the amputation. In the same medical note, she was diagnosed with partial deafness and moderate visual impairment. Later, in an obituary, Karl von Frisch would mention that her compromised health was the reason that she abandoned her original plan to pursue a career in agriculture (von Frisch 1960).

Throughout her adult life, the disability and other chronic health problems (such as partial deafness) seriously interfered with Ruth Beutler’s life and work. Memos written and signed by Karl von Frisch document two instances when Ruth Beutler had to take extended medical leave. On May 4, 1931, he requested that *Fräulein Dr. M. von Dehn* be appointed as a substitute of the ill *Fräulein Dr. Beutler*,^11^ thus indirectly indicating an extended medical leave of the latter. In a memorandum directed to the administrative office of the rector^12^ of the University Munich on May 7, 1943, he wrote:

*The health of the assistant at the Zoological Institute, Fräulein Professor Ruth Beutler, is currently severely compromised. According to the examination by Geheimrat Bumke (see attached), if feasible, she should be placed on medical leave. I request your approval for such leave from May 1, 1943, to October 31, 1943.*

*Heil Hitler!*^13^

*K v Frisch*^14^

The medical examination report signed by Professor Bumke provides more details:

*Fräulein Professor Beutler is so down that she should be relieved from her duties for a minimum of three months but, if possible, six months. This would enable her to work in the institute only as much as she feels comfortable with, given her current mental health condition.*^15^

## Science education and early scientific career

### Undergraduate studies and thesis research in comparative physiology

From 1919 to 1923, Ruth Beutler studied general science, zoology, chemistry, anthropology, and botany at the Universities of Jena, Leipzig, Munich, and Rostock.^16^ It was in Munich, in 1920, that she met Karl von Frisch and became his second doctoral student. When he moved to the University of Rostock, she followed him. There, in 1923, she successfully defended her PhD thesis entitled *Experimentelle Untersuchungen über die Verdauung bei Hydra* (Experimental Studies of Digestion in *Hydra*). It was published as the very first article in the *Zeitschrift für vergleichende Physiologie* (Beutler 1924).

The topic had been suggested by von Frisch and was of particular interest because, at that time, it was unclear whether *Hydra* still relied on intracellular digestion of nutrients (like unicellular organisms), or whether (like ‘higher’ metazoans), it predominantly exhibited extracellular digestion. Before Beutler’s study, researchers had almost exclusively carried out histological studies to address this question. By contrast, in what would become one of the hallmarks of comparative physiology, Ruth Beutler developed carefully designed physiological approaches based on extensive observations of the behavior of the animals to examine digestion in live polyps. These experiments demonstrated that cells of the endoderm of *Hydra* can absorb food particles by phagocytosis. At the same time, a degradation of proteins through proteolytic enzymes in the gastric region can take place, and then the dissolved proteins, or smaller fragments of them, are internalized by endodermal cells.

After her death, von Frisch commented:

*With her thesis research…she demonstrated methodological skills and scholarly spirit. Her findings were a valuable contribution to the comparative physiology of digestion (p. 10; *von Frisch 1960*)*.

### Expanding her expertise into physiological chemistry

After obtaining her PhD, Ruth Beutler had stints at the Zoological Institutes in Rostock and Breslau, at the Zoological Stations on the island of Helgoland and in Naples, as well as at the Institute of Physiological Chemistry at the University of Leipzig.^17^ At the latter, she broadened her education by undergoing a thorough training in physiological chemistry—something rather rare among zoologists at that time. In 1925, she returned to the Zoological Institute of the University of Munich to rejoin Karl von Frisch, who had just accepted the offer to succeed his former mentor, Richard von Hertwig,^18^ as *Ordinarius*.^19^

Even though Ruth Beutler had a PhD level of education, she was employed only as a lab technician.^20^ Nevertheless, she continued her scientific work, applying the newly acquired skills in physiological chemistry to the study of several biologically important phenomena. This work culminated in two lines of studies. The first was the most comprehensive investigation undertaken up to this point to determine the sugar concentration in the nectar of flowers of various plant species visited by honeybees; and to identify the different types of sugar (glucose, fructose, and saccharose) produced. As part of this work, she examined the effect of different biological conditions, such as humidity and moisture levels in soil, on the sugar concentration and composition of nectar. Her findings had implications not only for basic science but also for applied science, as they showed how honey production could be optimized using scientific approaches. The study was published in the *Zeitschrift für vergleichende Physiologie* (Beutler 1930), encompassing no less than 104 printed pages.

**Fig. 5:**
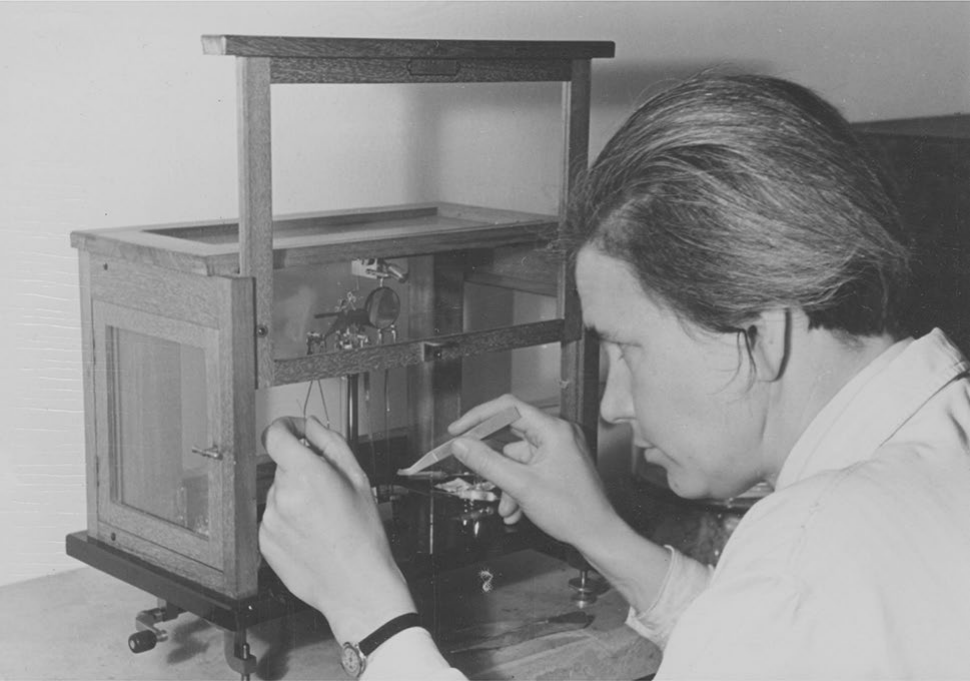
Ruth Beutler using a precision scale to determine the concentration of blood sugar. The picture was taken by the renowned photographer Georg Schödl on November 11, 1937, on the occasion of a radio broadcast titled *Geheimnisse der Bienenwelt* (The Secretive World of Bees). Courtesy: Stadtarchiv München (DE-1992-FS-PER-B-0308-01)

In her second line of research, Ruth Beutler focused on a comparative study of blood sugar in animals, with a particular focus on honeybees. Up to the time of her research, two key questions had remained unanswered: First, which organ stores glucose (a function assumed by liver and muscles in vertebrates)? Second, does a regulation of the blood glucose level take place, although a pancreas, with its insulin-producing β-cells in the islets of Langerhans, is absent from invertebrates? By developing a method for determining the blood sugar concentration in minute volumes (Beutler 1935), she was able to provide, for the first time, answers to these questions (Beutler 1936). Figure 5 shows her determining the concentration of blood sugar by use of a precision scale.

Employing the newly developed method, Ruth Beutler demonstrated that the concentration of glucose in the blood of bees is extremely high, about 20-times higher than the corresponding level in humans. Variations in blood sugar concentration correlate with the expected energy expenditure. For example, when a worker bee arrives with an empty honey stomach (an enlargement of the esophagus near the stomach) at a food source, she weighs about 70 mg and has a blood sugar concentration of approximately 1.3%. On the other hand, a worker bee with her honey stomach filled with nectar weighs about 110 mg and has a blood sugar concentration of approximately 2.2%. In the bee’s body, sugar is stored almost exclusively in the honey stomach, which, therefore, serves not only as an organ for transporting nectar to the hive but also as a storage site for replenishing blood sugar levels. If a bee is unable to refill the honey stomach, the blood sugar levels is depleted within less than 24 h and the bee will become exhausted.

### Habilitation

**Fig. 6:**
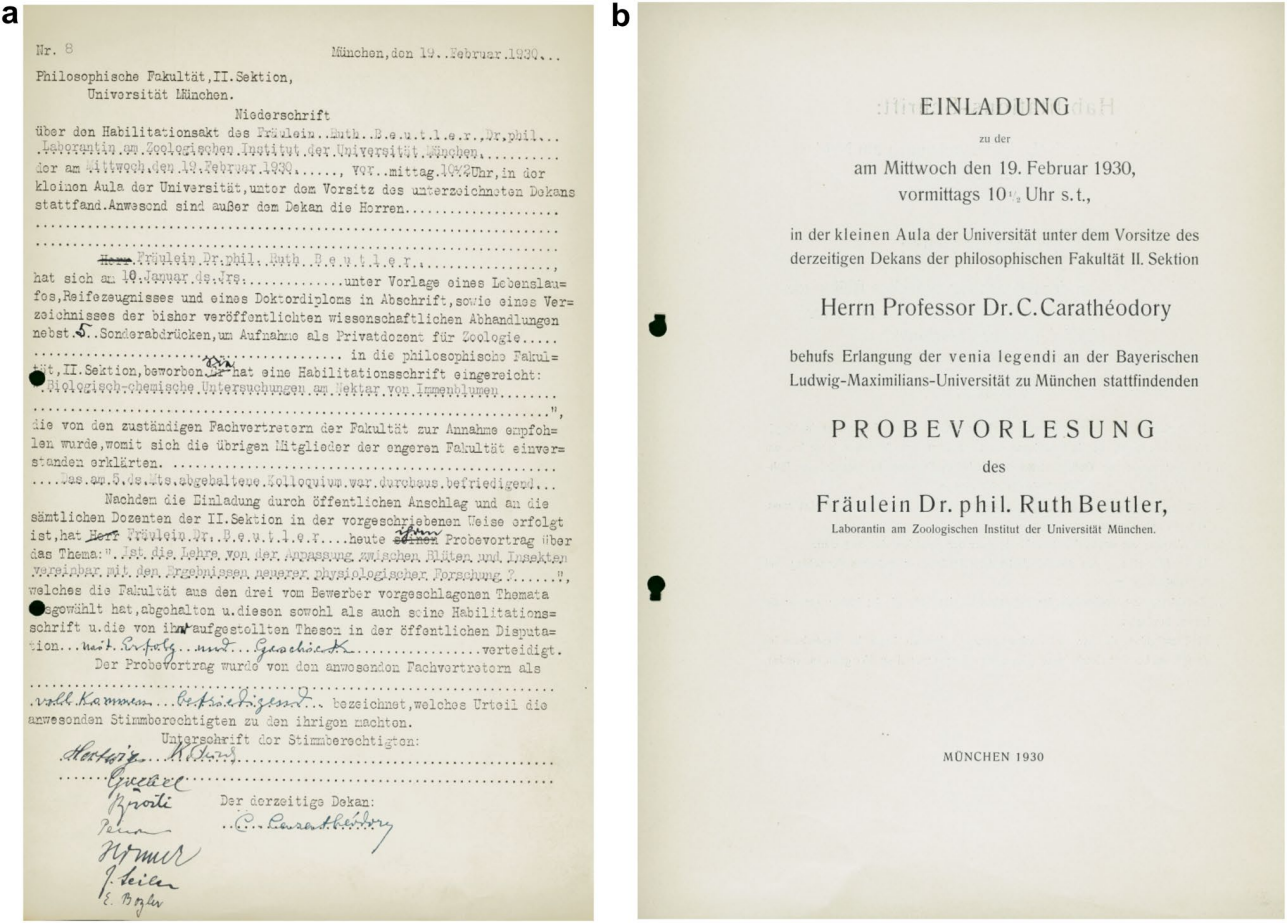
**a** Written record of the requirements fulfilled by Ruth Beutler for her *Habilitation*. The recommendation for the award of this academic distinction was made unanimously by the eligible faculty and the dean. Note that several masculine words related to the gender identity of the applicant on the printed form are crossed out and replaced by the corresponding feminine word. In one instance, the masculine possessive pronoun was forgotten to be replaced so that the text reads *seine Habilitationsschrift* (his *Habilitation* thesis) instead of *ihre Habilitationsschrift* (her Habilitation thesis). The two signatures in the first signature line are those of Richard von Hertwig and Karl von Frisch. **b** Invitation to the trial lecture of Ruth Beutler on February 19, 1930. The lecture was part of the requirements for obtaining the authorization to teach (*venia legendi*). The session was chaired by the dean of the Faculty of Science, Constantin Carathéodory. Courtesy: Universitätsarchiv München (FakBio-VII-7)

In 1930, Ruth Beutler received her *Habilitation* (the qualification required for appointment as professor at German universities) in zoology.^21^ Back then, she was only the second woman at the University of Munich who had been awarded this academic distinction.^22^ How unusual the *Habilitation* of a woman was at that time is evident from the written record that documents her fulfillment of the requirements for *Habilitation* (Fig. 6). On the form, only masculine words were printed in relation to the gender identity of the applicant, e.g., *Herr* (Mister), *er* (he), and *seiner* (his), reflecting the assumption that men only would apply for the *Habilitation*. Hence, on Beutler’s form these masculine words had to be crossed out and replaced by the corresponding feminine words, e.g., *Fräulein* (Miss), *sie* (she), and *ihrer* (her).

Despite her *Habilitation*, which came with the right to teach at the University of Munich in the area of zoology, she remained employed as a lab technician.^23^ Only in 1932, she was appointed as a *wissenschaftliche Assistentin* (research associate). Commenting on the inadequate level of her employment, later Karl von Frisch wrote:

*It is noted with gratitude, combined with expression of shame, that from 1925 to 1932, i.e., including two years after her Habilitation in 1930, she carried out the work of a research assistant while being employed as a lab technician at the Zoological Institute* (p. 11, von Frisch 1960).

## The Nazi era

### Ruth Beutler: proving Aryan descent and appointment as university lecturer

On January 30, 1933, President Paul von Hindenburg appointed Adolf Hitler as the new Chancellor of the *Reich*. Two months later, on April 7, 1933, the *Gesetz zur Wiederherstellung des Berufsbeamtentums* (Law for the Restoration of the Professional Civil Service) was signed into law.^24^ It forced civil servants who were not of Aryan descent to retire, initially with the exception of those who had fought at the front for the German Reich, or whose fathers or sons fell in World War I (Paragraph 3). The law also gave authorities the power to dismiss civil servants whose previous political activities afforded, under Nazi ideology, no assurance that they would, at any time, give their fullest support to the national state (Paragraph 4). The main, though not only, groups targeted by this law were Jews and communists.

To prove Aryan descent, members of certain professions, including university professors and *Privatdozenten* (unsalaried lecturers with the *Habilitation* qualification), had to fill in a form with information about themselves, their parents, and their grandparents, and to substantiate their claims by attaching corresponding documents from the civil registry offices and the parish offices. In a supplementary form, they had to provide information about their professional careers and possible memberships in political organizations.

Ruth Beutler submitted this mandatory questionnaire on June 3, 1933, claiming Aryan descent and providing information about her past membership in the DNVP.^25^ In an official list issued on July 18, 1933, the *Bayerisches Staatsministerium für Unterricht und Kultus* (Bavarian State Ministry for Education and Culture) confirmed that Beutler had successfully provided proof of her Aryan ancestry.^26^

In 1937, Ruth Beutler was appointed *nichtbeamteter außerordentlicher Professor* (comparable to a non-tenure-track adjunct professor) by the *Reich- und Preußische Minister für Wissenschaft, Erziehung und Volksbildung* (Minister of Science, Education, and National Culture).^27^ Her appointment was supported by the leader of the *Dozentenschaft* at the University of Munich, Wilhelm Führer.^28^ In an undated evaluation of Ruth Beutler, he wrote:

*Fräulein Dr. Beutler is a very empathetic and pleasant colleague of great character … There is no doubt that, overall, she is supportive of the ideology of national socialism … However, there are limits in terms of her political beliefs. In many ways, she is under the influence of Professor von Frisch, for whom she expresses a great deal of admiration. Therefore, she often goes along with him. Nevertheless, it is important to stress that Fräulein Dr. Beutler is one of the few pleasant characters of the Zoological Institute, thanks to her mindset that is pure and free of intrigues.*^29^

In his evaluation report, Führer did not indicate what made him believe that Ruth Beutler had a positive attitude towards national socialism. However, it is likely that Führer was aware of her membership in the DNVP. Given the ideological closeness of DNVP and NSDAP, he might (probably rightly) have assumed that Beutler was, at least to a certain extent and at that point of time, sympathetic to the Nazi movement.

On February 17, 1939, a new regulation for the *Habilitation* at universities of the *Reich* took effect. Up to this date, the authorization to teach (*venia legendi*) had been bestowed by the respective university. The new regulation transferred this right (and thereby more control over the selection of applicants) to the government. As a result, every university lecturer who was not an *Ordinarius* had to re-apply for this authorization. Karl von Frisch supported Ruth Beutler’s application for appointment as *Dozentin neuer Ordnung* (lecturer under the new regulation), which was finally successful. What is notable about his letter of recommendation is that it was stronger than any other I found in the archives.

*Frau Professor Ruth Beutler is my most successful staff member.*^30^* Her studies on comparative physiology of digestion, her biological-chemical investigations of nectar, and her research on blood sugar of animals are all of great significance and originality in terms of their methods. The results of this work are masterpieces. She is well-known and highly regarded beyond the Reich. Under her leadership, a large number of student projects have been carried out. As a university lecturer, she teaches an area within comparative physiological chemistry that is underrepresented, yet particularly interesting. She is, therefore, able to close a significant gap in our zoological curriculum. Her lectures cover topics comprehensively and vividly; they are very popular among students. I highly recommend her appointment to the post of Dozentin neuer Ordnung.*^31^

### Karl von Frisch: a quarter-Jew navigating the threat of forced retirement

Karl von Frisch signed the questionnaire related to the Law for the Restoration of the Professional Civil Service on June 13, 1933, indicating catholic fate of his parents and grandparents.^32^ Based on this form, the Bavarian State Ministry for Education and Culture acknowledged his proof of Aryan descent in the official list issued July 18, 1933.^33^

However, in a letter of April 6, 1936, addressed to the rector’s office, Wilhelm Führer, now acting on behalf of the *Nationalsozialistischer Deutscher Dozentenbund*^34^ of the University of Munich, requested von Frisch’s claim of Aryan descent to be re-reviewed, with high priority.^35^ This request was supported by testimonies signed on the same day by two assistants at the Zoological Institute of the University of Munich, Johannes Scharnke and Karl Eller.^36^ They testified that they had obtained information about the Jewish bloodline of at least one of von Frisch’s grandparents. In the same letter, and in a more detailed, eight-page-long letter dated May 15, 1936, to the *Reichsministerium für Wissenschaft, Erziehung und Volksbildung* (Reich Ministry of Science, Education, and National Culture), Wilhelm Führer and Ernst Bergdolt^37^ (acting on behalf of the *Nationalsozialistischer Deutscher Dozentenbund* and the *Dozentenschaft* of the University of Munich) condemned von Frisch as a patron of Jews and as an opponent of the ‘New Era’:

*The preference of Professor Frisch for anything Jewish, obviously based on his own Jewish blood, is compensated by a sometimes rather poorly hidden hate of national socialism*.^38^

This request to re-review von Frisch’s racial descent, and to replace him by a new professor and director true to the party-line, triggered over the next few years further inquiries by several authorities of the Bavarian government in Munich and the Reich government in Berlin. On January 9, 1941, Karl von Frisch received, through the rector’s office, the following letter of the Bavarian State Ministry for Education and Culture:

*“…according to the investigations of the Reich Ministry of Education, the Ordinarius at the University of Munich, Dr. Karl von Frisch, has been identified as a Mischling of the second degree.*^39^* Thus, the Reich Minister of Science, Education and National Culture intends to retire him early.*^40^

Karl von Frisch vehemently refused to accept the intended forced retirement or, as had been suggested to him, to request himself early retirement. In an undated memorandum, probably written shortly after he had received notification of the intended forced retirement, he emphasized that...

*“…through my teaching, my research work, and, last not least, my contributions to the public understanding of science, I have served Volk and Reich to the best of my ability. I am not aware that I have done anything, either in word or action, that conflicts with the goals of the national socialist government.”*^41^

During meetings with authorities at the Bavarian State Ministry for Education and Culture and the Reich Ministry of Science, Education, and National Culture, Karl von Frisch petitioned to reverse the intended forced retirement. He also made various efforts to demonstrate his loyalty that he had shown through his scientific work to the *Reich*. On February 2, 1941, he published a one-page article in *Das Reich*,^42^ in which he stressed the importance of the Zoological Institute not only for basic research and for teaching but also as a ‘feeder’ of applied science. At the end of his essay, he even went as far as praising the *Deutsches Reich* in its effort to utilize the work of contemporary genetics, “in the broadest form”, for the benefit of the Volk.^43^

The actions taken by von Frisch to prevent his imminent forced retirement were supported by several of his colleagues. Among the first, and perhaps most prominent, was Hans Spemann.^44^ On January 21, 1941, he wrote a letter to Bernhard Rust,^45^ the Reich Minister of Science, Education, and National Culture, in which he praised Karl von Frisch as “irreplaceable” and warned that the forced early retirement of von Frisch would be “the most severe blow to German biology one can think of.”^46^

The above petition letter and other expressions of support prompted discussions among high-ranking Nazi officials, including the Reich Minister of Science, Education, and National Culture, Bernhard Rust; the Reich Minister of the Interior, Wilhelm Frick^47^; and the Head of the Party Chancellery, Martin Bormann.^48^ However, possible considerations for granting exception to the forced retirement of Karl von Frisch were vehemently opposed by the leader of the *Dozentenschaft* of the University of Munich, Ernst Bergdolt, who argued that von Frisch...

*…neither is irreplaceable [as claimed by his supporters] nor, due to his political attitude, does he, in the slightest, deserve that the application of the law be waived*.^49^

Bormann, in a letter to Rust, came to the same conclusion:

*Before and after the* Machtübernahme *(political takeover by the Nazis), Professor von Frisch interacted extensively with Jews. He has also repeatedly emphasized that science has suffered significantly through the emigration of Jews. I have received reports that he tried to remove all scientific and non-scientific staff with antisemitic attitudes from the Zoological Institute.*^50^

Rust concurred with the recommendations made by Bergdolt and Bormann. In a letter dated October 2, 1941, he informed the Bavarian State Ministry for Education and Culture about his intention to enforce the retirement of Karl von Frisch.^51^

The end of von Frisch’s scientific career seemed now inevitable. At this point of time, it is unlikely that further expression of support alone would have changed Rust’s decision. However, around the same time help came from a rather unexpected source—the *Reichsministerium für Ernährung und Landwirtschaft* (Reich Ministry of Food and Agriculture). Between 1940 and 1942, beekeepers in Germany and other European countries suffered heavy losses totaling hundreds of thousands of bee colonies due to nosemosis. This disease is caused by the microsporidium *Nosema apis*.^52^ Infection with this parasite of adult *Apis mellifera* at a young age affects the digestive system and causes them to not produce brood food (royal jelly). These bees also frequently skip the brood-rearing stage and become forager bees right away. Infected queen bees cease laying eggs. In addition, both types of bees have a shortened lifespan. The combination of these effects can result in the death of the colony.

The loss of bees due to the nosemosis plague resulted in a substantial reduction of the production of honey. Yet, economically even worse was the damage done through reduced crop pollination. A nosemosis task force, headed by Franz Wirz,^53^ was established to coordinate and fund research for combating the disease. In his memoirs, without mentioning the name of Wirz, Karl von Frisch wrote:

*The chairman of this task force was an influential person in the area of food production. He was familiar with my work, and he was aware of my imminent forced retirement. He fervently acted on behalf of myself and the Institute. He deserves the credit that I was commissioned by the Reich Ministry of Food and Agriculture to carry out research on nosemosis* (p. 116; von Frisch 1957).

**Fig. 7:**
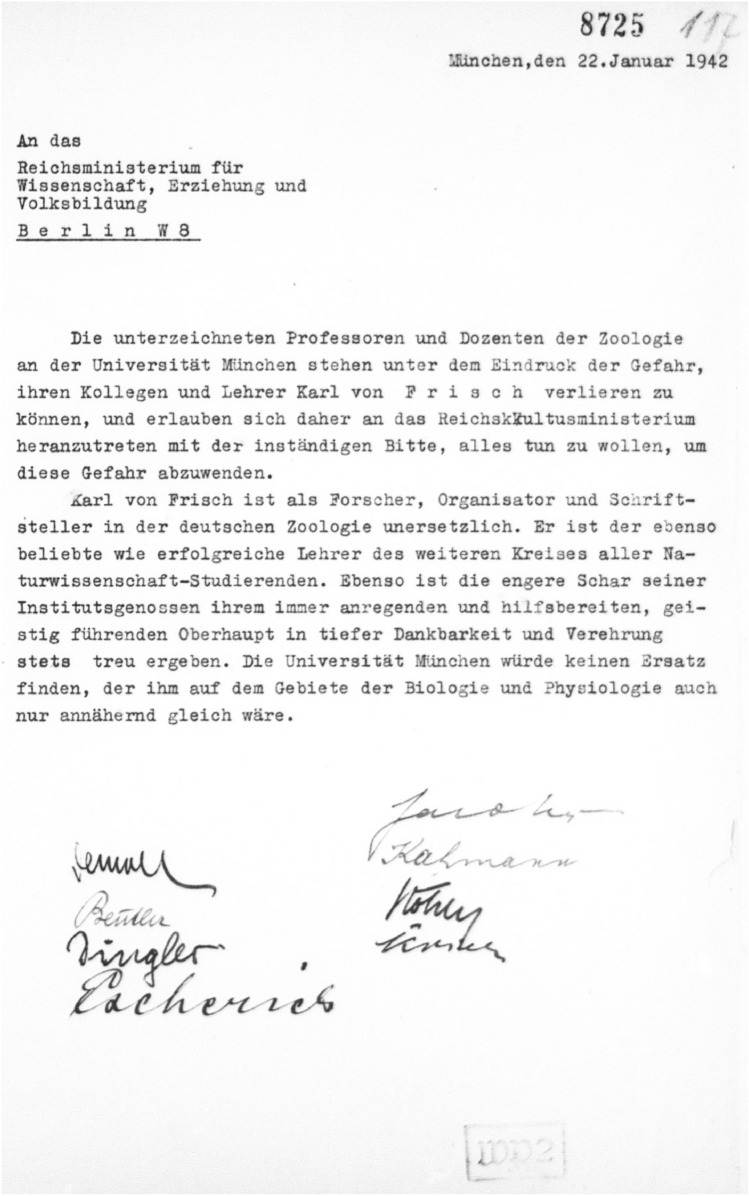
Petition letter to the Reich Ministry of Science, Education, and National Culture, signed by eight professors and lecturers of zoology at the University of Munich. In this letter, dated January 22, 1942, the signatories expressed their strong support of Karl von Frisch to prevent his forced retirement (for a translation of the letter see main text). The second signature on the left is that of Ruth Beutler. Courtesy: Bundesarchiv Berlin-Lichterfelde (R 4901/24579)

On October 23, 1941, upon the invitation of the Reich Minister of Food and Agriculture, Richard Walther Darrè,^54^ the nosemosis task force, chaired by Wirz, met. Karl von Frisch, who attended this meeting, was selected to coordinate the scientific efforts. Given von Frisch’s mastery in pulling the strings, it might have been no accident that soon after this meeting a petition letter arrived at the Reich Ministry of Science, Education, and National Culture, signed by eight zoology professors and lecturers at the University of Munich (Fig. 7). The signers included Ruth Beutler and the following:

Reinhard Demoll (1882–1960). He was *Ordinarius* for Zoology and Ichthyology at the Faculty of Veterinary Sciences and widely renowned for his scientific work; he served as rector of the University of Munich during the academic year 1931/32.

Max Dingler (1883–1961). He was a zoologist, Bavarian dialect poet, environmentalist, and director of the Bavarian State Natural Science Collection. Although regarded by many of his colleagues a rather mediocre scientist, he was bestowed an honorary professorship at the University of Munich, primarily based on his engagement for the Nazi movement (Litten 1996).

Karl Escherich (1871–1951). He was *Ordinarius* for Applied Zoology at the University of Munich, where he specialized in applied entomology. He was rector of the University from 1933 to 1935. An early member of the NSDAP, he participated in the Munich Beer Hall Putsch, a failed attempt led by Adolf Hitler to overthrow the German government in 1923.

Werner Jacobs (1901–1972). A highly recognized entomologist, he was a *Kurator* and *außerordentlicher Professor* at the Zoological Institute.

Herman Kahmann (1906–1990). A zoologist specializing on small mammals, he was a zoologist who had joined Karl von Frisch as an assistant in 1938. Tiefenbacher (1991), in an obituary of Kahmann, quotes the following comment made by von Frisch after World War II: “He [Kahmann] was a fierce opponent of national socialism. Often, he expressed his opposition so openly that he put himself into enormous danger.”

I was not able to unambiguously identify the signatures of two other signers. One might have been Wilhelm Köhler, a *wissenschaftlicher Assistant* (lecturer) of zoology and genetics.

The letter reads as follows:

*The signatories of this memorandum, professors and lecturers of zoology at the University of Munich, are extremely concerned of the imminent danger of losing their colleague and teacher Karl von Frisch. They, therefore, approach the Reich Ministry with the sincere request to do everything to avert this danger.*

*In German zoology, Karl von Frisch is irreplaceable as a researcher, organizer, and writer. He is a successful instructor who is much liked by students enrolled in a wide range of science degree programs. Likewise, the inner circle of fellows at his institute are eternally grateful to, and express their unrestricted admiration for, him, their intellectual leader. He is a constant source of inspiration and an example of unlimited willingness to help others. The University of Munich would not stand any chance to replace him; no one in biology and physiology even comes close to him.*^55^

It might have been Wirz’s repeated interventions in support of von Frisch,^56^ in combination with the help by other scientists, including the eight professors and lecturers of the University of Munich, that prompted Rust to revert his previous decision. On February 10, 1942, he granted permission that von Frisch could continue conducting his studies on nosemosis after his forced retirement.^57^ However, after von Frisch had sent a letter, dated March 14, 1942, to the Bavarian State Ministry for Education and Culture in which he argued that his retirement would severely limit his ability to do research, including the work on nosemosis,^58^ the Reich Ministry of Science, Education, and National Culture and Martin Bormann finally agreed to…

*…delay the retirement of Professor von Frisch until the time after the war.*^59^

Whereas the commissioned research on nosemosis protected von Frisch from forced retirement, it is unclear how much he himself engaged in this project. His bibliography, published by Autrum (1982), lists only two short communications on nosemosis (von Frisch 1943, 1944). In his memoirs, he spends just two lines on summarizing the outcome of this research:

*As a matter of fact, we did not succeed in bringing these studies [on nosemosis] to a satisfactory conclusion by the end of the war* (p. 116; von Frisch 1957).

It seems that most of the commissioned work was carried out by Ruth Beutler. Together with Elisabeth Opfinger, she published a paper on nosemosis (Beutler and Opfinger 1950). Nevertheless, even the results of their investigation were rather modest: feeding of pollen lengthens longevity of both healthy and infected young bees, but such a diet is effective only if it starts no later than on the tenth day of life of the bees.

### Making a key discovery in the midst of war: Karl von Frisch, Ruth Beutler, and the dance communication of honeybees

Karl von Frisch used a good part—probably most—of the remaining time until the end of the war for research that interested him far more than the nosemosis studies. At the same time, Ruth Beutler made an observation that, arguably, had a larger impact on von Frisch’s research than any other work of members of the scientific staff of the Zoological Institute. Her observations did nothing less than prompting Karl von Frisch to revisit an earlier idea that already had made him famous—how honeybees communicate, through their dances, sources of nectar and pollen to other bees of their colony.

**Fig. 8:**
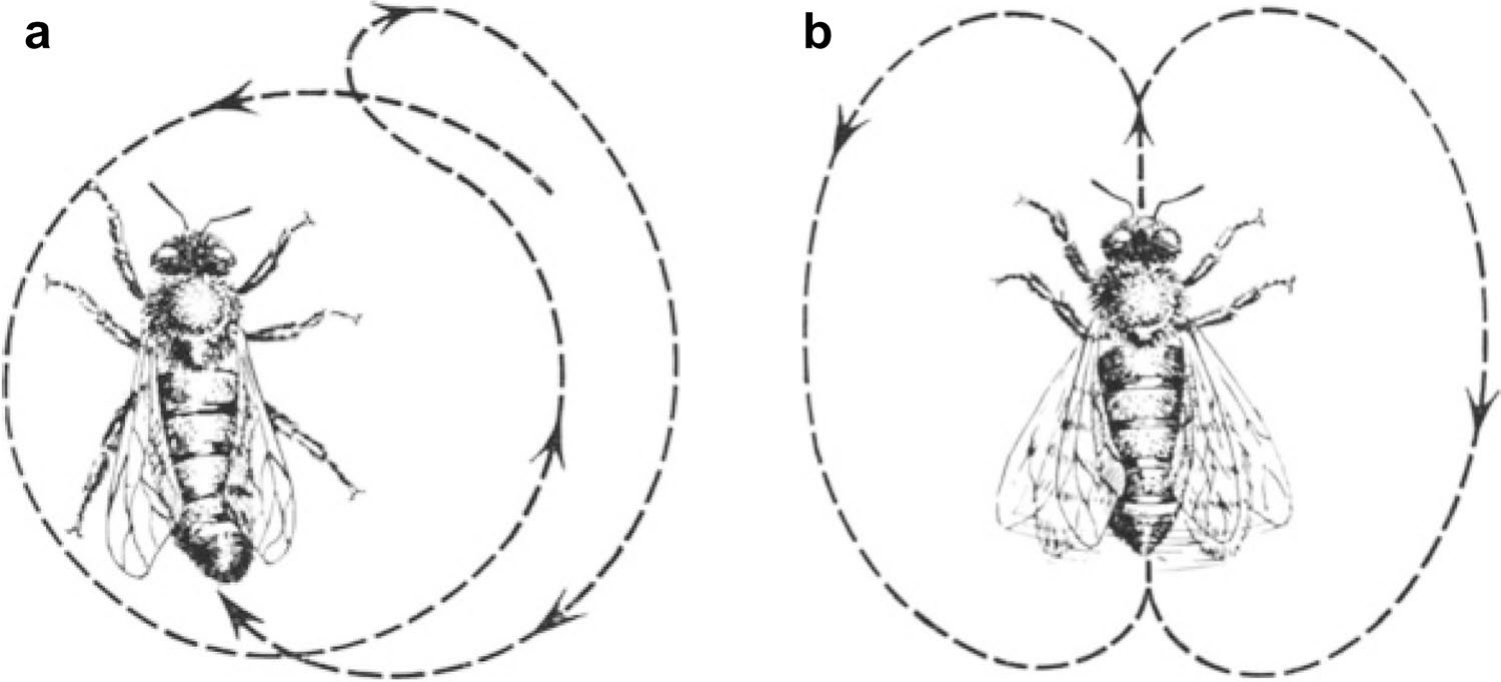
Dance communication in bees. **a** Round dance. **b** Waggle dance. In both figures, the dashed lines, in combination with the arrows, indicate the course taken by the running bee. As Karl von Frisch demonstrated in the study published in 1946 for the first time, round dances are used by the bees to indicate food near the hive, whereas waggle dances are displayed to communicate information about food at distances greater than approximately 50–100 m. The frequency of the straight runs in the waggle dance conveys distance information. Reproduced from von Frisch (1957). Copyright Springer-Verlag, Berlin/Heidelberg. With kind permission of Springer-Verlag

These two types of food are essential for meeting the energetic and nutritional demands of the colony—nectar as a source of carbohydrates, and pollen as a source of proteins and fat. In 1923, von Frisch reported that when a foraging bee discovers a profitable source of nectar (in experiments provided in form of a concentrated aqueous solution of sugar), it conveys this information to the other bees in the hive by performing a round dance (von Frisch 1923) (Fig. 8a). Later, he confirmed that the same behavior can be observed under natural conditions when bees collect nectar from flowers (von Frisch 1942). In both cases, potential foragers are attracted by this display so that they closely follow the dancer, thereby acquiring olfactory information associated with floral particles that became impregnated onto her body during nectar collection. These odors enable the recruited foragers to localize the specific food source from which the dancing bee has collected the nectar (or the sugar solution in a petri dish placed on a scented filter paper).

According to von Frisch’s hypothesis published in 1923, bees that collect pollen also transfer information about the food source to potential foragers, yet they exhibit a different behavior, the waggle dance (Fig. 8b). In the latter case, the advertised food source is specified by the odor associated with the pollen carried on the hind legs of the incoming foragers. In an expanded version of the original hypothesis, von Frisch and his student Gustav A. Rösch interpreted the tail wagging during the straight-run portion of the waggle dance as a mechanism to catapult pollen from the hind legs onto the olfactory organ on the antennae of the closely following bee (von Frisch and Rösch 1926).

The proposal of the differential roles of the round and waggle dances of bees remained largely unchallenged until Ruth Beutler made (the exact date is unknown but probably around 1944) a surprising observation. Karl von Frisch himself provides a brief account of this event:

*I was prompted to revisit the phenomenon of bee communication after a conversation with my assistant of many years, Professor R. Beutler. As part of a research project, she wanted to work with bees at a far distance from the hive. To avoid the time-consuming movement of a petri dish with the sugar solution, I had suggested establishing a feeding place in the closer vicinity to the hive. Recruited foragers would then soon appear at the distant feeding place. Yet, this approach failed. This and other observations indicated that it is possible that bees can communicate the distance of a food source from the hive*^60^ (p. 3, von Frisch 1946).

In the summer of 1944, Karl von Frisch, therefore, began at his country estate in Brunnwinkl (for a historical account of this family estate and its importance for Karl von Frisch’s research see von Frisch 1980) to re-examine the functional significance of the bee dances in communicating food sources. During this and the following year, he probably made the most significant discovery of his scientific career, published in 1946 in the *Österreichische Zoologische Zeitschrift* (von Frisch 1946). It was this critical piece that firmly established his fame as the discoverer of the ‘dance language’ of bees.

The core idea communicated in this paper was that the two types of dances do not, as he had previously assumed (von Frisch 1923), specify different types of food. Instead, according to the revised hypothesis, they are used for indicating food sources at different distances to the hive—the round dance for food near the hive, the waggle dance for food at distances greater than approximately 50–100 m. In addition, the waggle dance conveys directional information.^61^

In support of this notion, Karl von Frisch presented a quantitative analysis based on hundreds of individual experiments he had conducted. The results indicated that the frequency of the straight-run portion of the waggle dance is inversely related to distance, whereas the orientation with respect to the force of gravity of the dancing bee during the straight-line course encodes the angle between the food source and the solar azimuth as seen from the hive.

Karl von Frisch himself provided an explanation why he had misinterpreted the significance of the round and waggle dances in his 1923 paper. Back then, he always had conducted feeding experiments using sugar solutions near the hive. Upon returning to the hive, these bees displayed round dances. On the other hand, under the experimental settings, foraging bees carrying pollen had, most commonly, collected them at greater distances from the hive. Consequently, they exhibited waggle dances to recruit hive mates.

### Bombing of the Zoological Institute

After the Allied invasion of Italy in September 1943, the aerial bombing attacks on Munich intensified. During an air raid carried out by the United States Army Air Forces on July 12, 1944, the house of the von Frisch family in Harlaching, a municipality in the southwest of Munich, was struck by two high-explosive bombs and reduced to rubble. During the next day’s air raid, the Zoological Institute was severely damaged.

*Now it was almost impossible to do research in a well-organized manner* (p. 123, von Frisch 1957).

After these events, Karl von Frisch stayed most of the time in Brunnwinkl and went to Munich only for short visits. He was temporarily joined by many of his staff, who used two houses of his estate as a ‘satellite laboratory.’

## Rebuilding the Zoological Institute

Shortly after the end of World War II, in 1946, Karl von Frisch accepted the offer to join as *Ordinarius* the University of Graz in the Southern Austrian province of Styria. In his memoirs, he defended the decision to retire from his position at the University of Munich and resume his work at the University of Graz:

*The situation in Munich was appalling. I was convinced that the reconstruction of the institute would take longer than the time that was left until my retirement. Our residence was a heap of rubble. The mere thought of Munich revoked painful memories of all the nightmare of the previous decade. I was hoping that a change of environment would provide the peaceful atmosphere that scholarly work needs to flourish … Then, at the age of 60 years, I was confronted with two alternatives: to rebuild the Zoological Institute in Munich, or to devote my time to research in Graz. I opted for the latter. Ruth Beutler, my very capable assistant of many years, was in charge of what had been left of the former Munich laboratory. With her never-ending enthusiasm, her intention was to make the institute operational again so that I would return from Graz. However, I had little intention to do so. I regarded my departure from Munich as final. Nevertheless, she remained determined to work towards this plan, until I, indeed, returned to Munich (pp. 131–132, *von Frisch 1957*)*.

Although Beutler was in charge of the Zoological Institute after Karl von Frisch had left Munich (officially, he retired from his post on September 30, 1946; however, he had been on extended leave of absence during most of the previous two years), she expressed her frustration about the conditions under which she had to manage what was left of the staff as well as the research and teaching facilities.

*I feel it is unjust that other professors who act as interim institute directors on behalf of their PIs (several of them, such as Maucher*^62^* from Geology and Souci*^63^* from Pharmacy, are even much younger than I am) receive the pay and have the rights of an Ordinarius, while Suessenguth*^64^* and I do not have such privileges. Recently, I asked the Dean to help the two of us just a bit in this matter, but, unfortunately, C.*^65^* didn’t take any action, out of personal reasons*.^66^

Ruth Beutler’s difficult financial situation in the years after the war was worsened by the fact that her father, who had supported her previously, died in 1942, and the family lost their estate in Thum in 1945/1946. In addition, she accommodated in her apartment in Munich her nephew Christian Schmidt, after his mother, Ruth’s sister Brigitte, had passed away. She was, therefore, grateful that Karl von Frisch had sent a letter of support to the Bavarian State Ministry for Education and Culture but added:

*I don’t think that such a letter will help.*

Obviously, Beutler and von Frisch had discussed her urgent need for financial support previously because at the end of the letter she writes:

*When you say that I should have had a frank conversation with you a long time ago, then you might be right. However, naturally, one prefers that the other person takes notice and approaches you, instead of you having to impose yourself. You cannot appreciate something like this because you probably have never been in such a situation … In any case, now I must take care of myself, and I beg you for help.*

Given von Frisch’s status, it is likely that his letter of support had weight, although it is unclear whether it was his intervention that finally led to Beutler’s appointment as Interim *Ordinarius* of Zoology and Comparative Anatomy in the Faculty of Science of the University of Munich. Interestingly, in the initial dispatch from the Bavarian State Ministry for Education and Culture, dated January 26, 1948, the (retrograde) appointment was effective September 1, 1947.^67^ However, three weeks later, on February 13, 1948, for reasons unknown, the Ministry revised its previous decision and made the position start six months earlier, on April 1, 1947.^68^

Nevertheless, neither of the two appointment letters, besides specifying her salary, mentions any funding for support of additional staff or research work. As evident from the correspondence between the Faculty of Science and the Bavarian State Ministry for Education and Culture, as well as the letters exchanged by Ruth Beutler and Karl von Frisch, after his official retirement in 1946 the main task of her was to prepare the Zoological Institute for a newly appointed *Ordinarius*. I have not found any document indicating that she was, at any point of time, considered as von Frisch’s successor.

However, what becomes clear from multiple documents is that, despite the retirement from his post in Munich, it was Karl von Frisch who continued to pull the strings from Graz and Brunnwinkl. Ruth Beutler kept him informed in letters about the outcome of negotiations with candidates and of discussions within the University. The search committee for identifying his successor included himself, Fritz Machatschek,^69^ and Karl Suessenguth.

Their initial list of potential candidates sent to the Bavarian State Ministry for Education and Culture on May 20, 1946, consisted of only three names, Paul Buchner,^70^ Otto Koehler,^71^ and Wilhelm Goetsch.^72^^,^ 73 Buchner declined the invitation on March 24, 1947.^74^ Koehler seemed to have received a rather hostile reception when he visited the University of Munich. He describes these hostilities in considerable detail in a letter to the Bavarian State Ministry for Education and Culture, dated June 14, 1948.^75^ Ruth Beutler also reports this hostile atmosphere in several letters to Karl von Frisch, who was not present during Koehler’s visit to Munich. The unfriendly reception might have impacted Koehler’s considerations. In a letter to the Ministry, instead of directly declining the offer, he writes that…

*…if Karl von Frisch accepts the Ordinarius position in Munich, then I will decline the offer.*^76^

Koehler’s letter prompted Professor Hans Rheinfelder of the Bavarian State Ministry for Education and Culture to send a letter to Karl von Frisch in which he writes:

*Although Professor Koehler has not declined the offer, he has stressed time and again that he would welcome it if you returned to Munich to resume your Ordinarius position. Then, he would be happy to step down as a candidate. A critical condition for your return has been that you will receive a permanent permit for travel between Austria and Bavaria.*^77^* Unfortunately, several attempts to obtain such a permit on your behalf have failed. Are you prepared to waive this condition and return to Munich without such a permanent permit?*^78^

Karl von Frisch replied on August 5, 1948:

*Making a decision has rarely been as difficult as this one but I definitely decline your kind request of my return to Munich. Of all the conditions I had listed previously as a prerequisite for entering negotiations not even a single one has been fulfilled: neither the permanent permit nor the guarantee of foreign currency for Austria nor my relief from teaching during the summer semesters. Your assurance to continue your efforts in this matter is not sufficient because I am profoundly rooted in Austria. I would not like to take the risk of being cut-off from my home country.*^79^

Seven days later, Otto Koehler rejected the offer to succeed Karl von Frisch as *Ordinarius* and Director of the Zoological Institute.^80^

In the meantime, Albert Maucher had become Dean of the Faculty of Science. He was immediately faced with the dilemma that the top two candidates from the list of three had declined the offer to head the Zoological Institute, and the third, Wilhelm Goetsch, was already over 60. Thus, in a letter to the Bavarian State Ministry for Education and Culture, dated October 15, 1948, Maucher requested to remove Goetsch from the initial list and approve a new list, with Erich von Holst^81^ as the top candidate, followed by Werner Jacobs and Anton Koch (a student of Paul Buchner).^82^ Karl von Frisch endorsed von Holst and Jacobs in a letter to the Bavarian State Ministry for Education and Culture, dated November 16, 1948.^83^ However, von Holst declined the offer on September 13, 1949.^84^

By then, the position had been vacant for three years. The potential prospect of further delays may have been the reason that Otto Jessen,^85^ then Dean of the Faculty of Science, made a rather surprising move, which is conveyed in a letter to the Bavarian State Ministry for Education and Culture.

*In a meeting on January 11, 1950, the Faculty of Science unanimously decided to suggest Professor Karl Ritter von Frisch for the position of Ordinarius of Zoology at the University of Munich.*^86^

From then on, matters moved quickly. The Ministry made the offer to Karl von Frisch on January 27, 1950. He received the offer letter on February 6, 1950, and on the same day he replied:

*In principle, I am delighted to return to my previous Chair position in Munich.*^87^

**Fig. 9:**
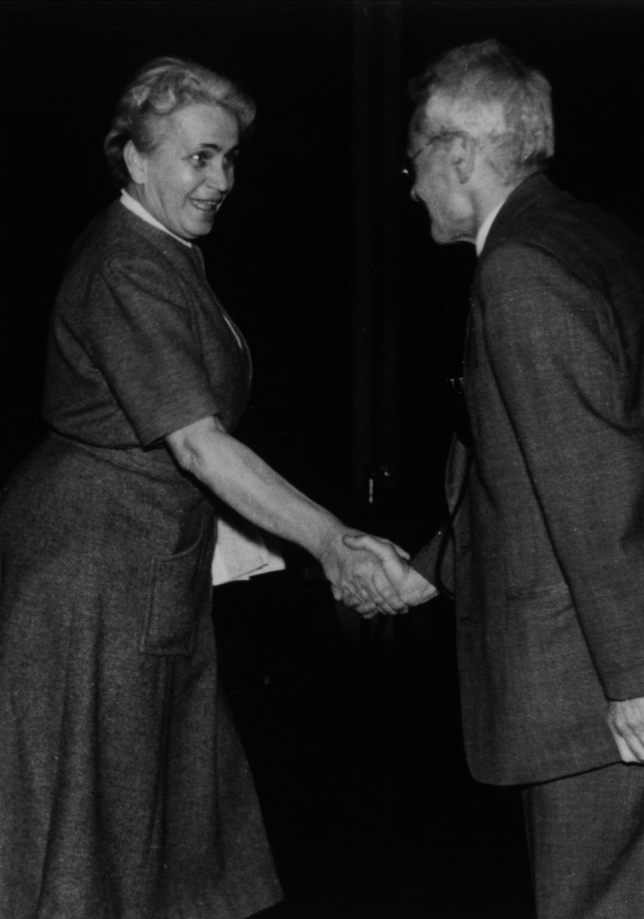
Ruth Beutler and Karl von Frisch, probably in the 1950s. The photographer and the occasion at which this picture was taken are unknown. Since the copyright term for *Lichtbilder*, such as images captured by photojournalists, in Germany is 50 years after their creation, this photograph is in the public domain

This time, the negotiations between Karl von Frisch and the Bavarian State Ministry for Education and Culture were successful. He returned to his former position with the University of Munich on May 1, 1950. Figure 9 shows Ruth Beutler and Karl von Frisch around that time.

Four years later, when he reached the compulsory retirement age, he was forced to retire but continued as Interim *Ordinarius* and Director of the Zoological Institute until his successor, Hansjochem Autrum,^88^ became the new head of Zoology in 1958.

While Karl von Frisch served as Interim *Ordinarius*, he published his memoirs. In them, he writes that, besides a larger pool of capable PhD students and a better funding situation,…

*…a decisive factor [to return] was that the Institute in Munich had become operational again much faster than I had thought* (p. 154; von Frisch 1957).

The re-establishment of Zoology in Munich was not only an enormous challenge in the wake of all the damage done to the building by the war but was also met with substantial resistance from internal competitors. Since the Institute of Chemistry, which was located immediately adjacent to the Zoological Institute, had been completely destroyed by the end of the war, Karl von Frisch offered Heinrich Wieland,^89^*Ordinarius* of Organic Chemistry and Director of the Institute of Chemistry, that the chemists could be temporarily accommodated in the rooms of the Zoological Institute. However, according to von Frisch’s account…

*…the chemists developed a treacherous imperialistic attitude—they wanted to take over the entire building of the Zoological Institute by moving us into the Institute of Botany* (p. 132, von Frisch 1957).

Hansjochem Autrum, in his memoirs, used similar harsh rhetoric by calling the attitude of the Chemistry faculty towards Zoology an act of parasitism (p. 157, Autrum 1996).

One of the actions undertaken by Ruth Beutler to prevent the takeover of the Zoological Institute by the chemists from happening is documented in a letter, dated April 25, 1946, which she sent to Klaus Clusius, Dean of the Faculty of Science:

*When I went to the Bavarian State Ministry for Education and Culture, I learned from Geheimrat* [Privy Councilor] *Kollmann and Geheimrat Devoll that during the last faculty meeting a decision was made to take away from us the entire institute. On behalf of Professor von Frisch, I vehemently oppose this plan…*^90^

In one of her letters to Karl von Frisch, she describes how the already difficult negotiations of the allocation of space were further complicated in her dealings with Wieland because he…

*…does not regard me as an equal party.*^91^

However, Beutler’s perseverance paid off and her success is acknowledged by von Frisch in his memoirs:

*That this [the planned complete takeover by the chemists of the Zoology building] remained unsuccessful, and instead a situation prevailed [temporarily] that allowed the chemists to use half of our building for their purposes, is the merit of Ruth Beutler, thanks to her courageous representation of our rights* (p. 132, von Frisch 1957).

Internal communication within the Bavarian State Ministry for Education and Culture documents that the Bavarian government was well aware of the refusal of Wieland to return the rooms that the chemists had been granted only temporarily.^92^ Since the officials of the Ministry did not foresee an amicable settlement of the dispute, they decided that Chemistry had to gradually return the rooms. In the aftermath of what Wieland perceived as an unfair decision, he submitted his resignation, a move that even made news in the national press.^93^

## Final years and late recognition

Karl von Frisch had successfully negotiated a *Konservator* position for Ruth Beutler as part of the condition for his acceptance of the offer to return to Munich.^94^ Her appointment became effective beginning of 1951 and included *Berufung in das Beamtenverhältnis auf Lebenszeit*, i.e., a permanent position as a state employee.^95^ This employment status provided, for the first time, job security — something particularly important given her fragile health condition.

During these years, she continued to publish, although at a slower pace and primarily review articles. These publications included two original papers in the *Zeitschrift für vergleichende Physiologie*. In the first article (Beutler and Opfinger 1950), she and Elisabeth Opfinger reported the results of their work on nosemosis, as mentioned above. In the second paper (Beutler 1954), she examined the maximal distance that foraging bees travel from the hive to collect nectar and pollen, and how this distance is affected by biological factors and weather conditions.

Towards the end of her life, she worked on what would ultimately constitute a monumental comparative review of food and digestion. When she passed away on October 22, 1959, after a battle with cancer, the manuscript was in an advanced stage but still unfinished. As done so often before, she expressed her respect for, and loyalty to, Karl von Frisch, this time by dedicating the paper to him on the occasion of his 70th birthday (which he had celebrated three years earlier). Based on the last manuscript version, the review was published seven years after her death in the form of a book chapter comprising 312 printed pages (Beutler 1966), reflecting in terms of both content and length her extraordinary expertise in physiological chemistry.

The last sentence of this chapter (although perhaps not intended as such) reads like one of the pillars of her legacy: “It appears to be necessary to study animals not just in laboratory settings but also in their natural habitat over extended periods of time and under different conditions” (p. 970, Beutler 1966).

**Fig. 10:**
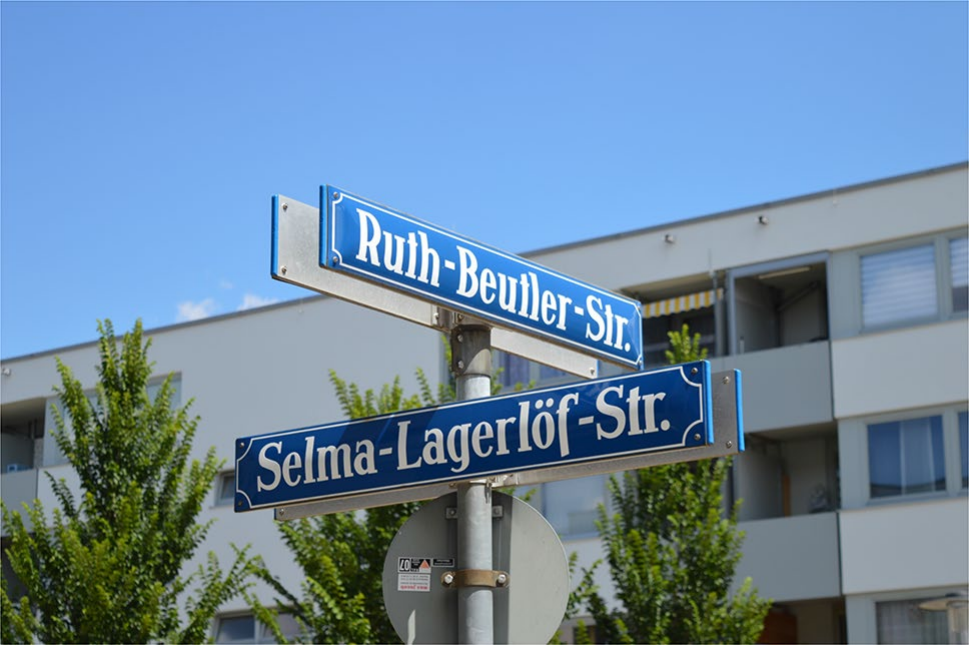
Ruth Beutler Street in Trudering-Riem, a municipality of Munich. It intersects with Selma Lagerlöf Street, named after the Swedish writer and Nobel laureate. Photograph by G.K.H. Zupanc

In 2004, the city of Munich named four streets in a new development area in the Trudering-Riem municipality after distinguished women. One of them is *Ruth-Beutler-Strasse* (Fig. 10), in recognition of Beutler’s physiological research on bees and her contributions to the reconstruction of the Zoological Institute of the University of Munich.^96^

## Ruth Beutler and Karl von Frisch: a hierarchical relationship dominated by unlimited loyalty and admiration

Although Ruth Beutler and Karl von Frisch had known each other for about four decades, nothing has transpired into today’s generation about the kind of their personal relationship. In none of the repositories in Munich and Berlin, I found any document dated earlier than 1944 that was exchanged directly between the two of them. However, after von Frisch moved most of his research operation from the University of Munich to Brunnwinkl in 1944 and retired from his *Ordinarius* position in 1946 until his return to Munich in 1950, a significant portion of their communication took place through letters. They are preserved in the archives of BSB (although the collection is clearly incomplete, as frequent reference is made to letters not present). In total, they comprise 23 letters written by Karl von Frisch between December 22, 1944, and April 15, 1950; and 28 letters written by Ruth Beutler between December 5, 1945, and October 21, 1950. This correspondence provides some indication of their personal relationship.

In all the letters, they addressed each other with the formal *Sie*, as opposed to the informal *Du*. However, the formal *Sie* was widely used in German by adult people of their generation, including acquaintances and even friends, and thus reveals little about their personal relationship. What is more indicative is how they addressed the other in the greeting line. Ruth Beutler always writes *Lieber Herr Professor* (Dear Professor), except in a few instances—after von Frisch had agreed to return to Munich—when she salutes him as *Lieber Herr Chef* (Dear Chief). Karl von Frisch, on the other hand, always addresses her as *Liebes Frl. Beutler* (Dear Miss Beutler), without including any academic title, such as ‘Doctor’ or ‘Professor’. Clearly, their personal relationship remained hierarchical, even though during most of these years she was Interim Director of the Zoological Institute and, thus, formally equal to him.

As evident from the actions Ruth Beutler took, particularly during the Nazi era and the years after World War II, she exhibited an enormous degree of loyalty towards Karl von Frisch. After his departure from Munich, she was obsessed with persuading him to return to Munich. As part of this goal, her core mission became the preparation of the Zoological Institute as an adequate research site, first (at least officially) for a potential successor of his, but soon for his own homecoming. She articulated this goal very clearly in 1946 when she published a piece in the journal *Naturwissenschaften* on the occasion of Karl von Frisch’s 60th birthday (Beutler 1946). (At that time, such public ‘birthday gifts’ were common in some scientific journals.) Besides highlighting his scientific accomplishments, she expressed her wish *that the separation from his old friends and coworkers will cease* (p. 191, Beutler 1946). At the end of the article, she comments on von Frisch’s character, something that equally illuminates her unlimited admiration for him:

*As a person, Karl von Frisch is not accessible for everyone. Yet, to those he feels close he presents himself without restraints. Vanity and outer seeming are foreign to him, but in his openness to true values he is enthusiastic like a young man. A fanatic when it comes to peace and protection of cultural goods, he suffered more than many others in that part of Europe that had been devastated by two world wars. We sincerely wish that his beloved home country [Austria], saved by a blessed fortune, will provide a safe and peaceful haven for his work as a researcher and teacher. That this wish comes true is the only comfort we can ask for, given the irreplaceable loss we suffered when he, a victim of World War II, moved away from us* (p. 192, Beutler 1946).

This article, which she had sent to Karl von Frisch on June 22, 1946, prompted the exchange of two letters, which are perhaps the most personal ones that I found in the collection at BSB. On July 5, 1947, he wrote:

*The day before yesterday, when I arrived [in Brunnwinkl], I found your letter of June 22nd, in addition to the reprint of your birthday gift published in ‘Naturwissenschaften’. I had learned about this piece three days earlier through a transcript that Gercken had arranged and sent to Graz. Luckily, I was on my own, so no one could watch me crying silently. Yet, it is you with whom I can share that your essay had exactly this effect. Considering that at such opportunities the good is stressed while the bad is ignored, you have plucked strings that, although they stopped producing sound, will swing very softly forever. You have done this in a wonderful way, you good old friend! Please accept my heartfelt thanks!*^97^

Ruth Beutler responded on July 20, 1947:

*Thank you so much for your lovely letter of July 5*^*th*^*. I got upset when I read that my article had saddened you. On the other hand, writing the article has made me, too, very depressed. It’s a shame that we can’t be together anymore. All the craziness of the world is reflected by this situation. It is self-evident that I have always regarded you as one of the most outstanding human beings I have met. Or do you think one bends over backward for another person for twenty years without good reason? I still haven’t given up hope that our paths will cross again — some day at some place. Europe must come to reason at one point.*^98^

## Epilogue

One hundred years after the publication of Ruth Beutler’s first scientific paper (Beutler 1924) and 65 years after her death, the picture that emerges of her is rather complex. The resources available through her family gave her access to an education that hardly anyone could have acquired who had not come from such a privileged family, especially if this person had a disability as severe as hers. Combining her education with outstanding experimental skills and a high degree of motivation and perseverance, she was one of the first women in Germany who was awarded the *Habilitation*. She was also one of the first scientists who applied methods from physiological chemistry to the study of zoological phenomena. As interim *Ordinarius*, her efforts, under the most challenging conditions of the post-war years, were instrumental in rebuilding the Zoological Institute.

Despite the highest academic qualification and her outstanding research record, she was, for several years, only employed as a technician. And when the names of potential successors of Karl von Frisch were discussed, and several of them were offered the vacant *Ordinarius* position, her name was never mentioned. Nevertheless, it is unclear whether Ruth Beutler had any ambitions to become *Ordinarius*, either in succession to von Frisch in Munich or somewhere else.^99^ As mentioned above, her priority was to devote all her energy to rebuilding the Zoological Institute so it would be attractive enough for him to accept the (repeated) offers to return to Munich—a goal in line with her undivided loyalty to him. Moreover, during her lifetime, women in *Ordinarius* positions were extremely rare, and their appointments were often met with fierce resistance. This reality might have discouraged many women to pursue an academic career, especially if it involved a leadership role. It was, indeed, only in 1923—the year Ruth Beutler received her PhD—that the first woman in Germany was appointed to an *Ordinarius* position.^100^ Ruth Beutler herself complains in her correspondence with von Frisch about her difficulties as interim *Ordinarius* to be treated equally by her Munich colleagues. Yet, in no instance does she imply that this reflects a disregard of her male colleagues toward women in general. Likewise, I have not found any indication that Beutler sympathized with the women’s rights movement. However, lack of support for feminism was not uncommon among women in science, engineering, and mathematics at her time, especially if their political views aligned with the ideology of conservative or fascist parties (Hänztschel and Bußmann 1997). It seems that Ruth Beutler saw her role primarily as that of the woman behind Karl von Frisch—to support and protect him whenever necessary, while accepting him as her superior and intellectual leader.

Ruth Beutler’s supportive and protective role was particularly important during the Nazi era. It is quite clear that she carried out most of the commissioned work on nosemosis—a project that provided little scope for noteworthy scientific achievements and whose sole purpose was, at least from von Frisch’s point of view, to save him from forced retirement. Through this arrangement, he was able to devote his time to a project that was at the core of his research interests and that is inextricably linked to his name—the dance language of honeybees. And it was Beutler’s observation that prompted him to revisit this phenomenon.

Would Karl von Frisch have discovered the true significance of the round and waggle dances without the failed attempt of Ruth Beutler to attract bees to the distant feeding place, using the approach he had suggested to her? In an essay about Ruth Beutler as a “female professor in the shadow of her male mentor” (subtitle of the article), Nagler-Springmann (1997) made the claim that Beutler was the skilled experimental worker doing the legwork for Karl von Frisch, while he, assuming the role of the scholar, used her ideas and research work for the establishment of his theoretical models, including the dance language of bees. Whereas the author cites *Biologen unter Hitler* (Deichmann 1992) in support of her thesis, what the latter book actually does is stating (correctly) that “Beutler’s observation led von Frisch to return to the study of bee communication” (p. 41).^101^ Besides failing to provide any further evidence, Nagler-Springmann (1997) apparently is also not aware of the fact that von Frisch was a very skilled experimenter who himself ran hundreds of field experiments to collect the evidence that led to the revision of his 1923 model.

Based on the historical evidence, the question is not who deserves the credit for the revision of the model. Rather, the question is whether Karl von Frisch would have returned to the phenomenon of bee communication through dances if Ruth Beutler had not provided the necessary motivation to do so. While there is no definite answer to this question, it is possible that, sooner or later, he would have performed experiments similar to the ones carried out in 1944 and 1945. When he mentions the conversation with Beutler that triggered these experiments, he writes that it was *dieser Umstand und andere Erfahrungen* that led him to suspect that bees, somehow, are able to communicate the distance of a food source from the hive (p. 3, von Frisch 1946). While *dieser Umstand* clearly refers to Beutler’s observation, *andere Erfahrungen* indicates additional evidence (without providing any further details) that was in conflict with his hypothesis proposed in 1923. Thus, perhaps the most plausible conclusion is that her observation has accelerated the establishment of the ‘dance language’ model by prompting von Frisch to re-test his original model earlier than he would have done otherwise.

## Data Availability

Data sharing not applicable to this article as no datasets were generated or analyzed during the current study.

## Abbreviations

BAB: Bundesarchiv Berlin-Lichterfelde
BayHStA: Bayerisches Hauptstaatsarchiv
BSB: Bayerische Staatsbibliothek
DNVP: Deutschnationale Volkspartei
NSDAP: Nationalsozialistische Deutsche Arbeiterpartei
StAM: Staatsarchiv München
StdA: Stadtarchiv München
UAM: Universitätsarchiv München

## Footnotes

1 Ritter (‘knight’) denotes the second-lowest rank within the German nobility.

2 Karl Ritter von Frisch (1886–1982) was an Austrian zoologist. In 1925, he was appointed *Ordinarius* at the University of Munich. In 1932, he received from the Rockefeller Foundation 993,000 Reichsmark (approximately 4.2 million U.S. Dollar, corresponding to a purchasing power of nearly 90 million U.S. Dollar in 2022) to build what was then the most modern zoological institute in Germany. In 1973, Karl von Frisch received the Nobel Prize in Physiology or Medicine for his pioneering contributions to the study of animal behavior. He was also co-founder of the *Zeitschrift für vergleichende Physiologie* (the predecessor of the Journal of Comparative Physiology A) and served as its Editor-in-Chief from 1924 to 1960.

3 Alfred Kühn (1885–1968) was a German zoologist and developmental biologist.

4 Sibylle Köhler (*née* Beutler), born July 27, 1936, is the daughter of Otto Beutler and his second wife Jutta Freiin von Wagner, and the niece of Ruth Beutler. Interviews were conducted by GKHZ at her residence in Kranenburg (Germany) on September 10, 2022, and January 4, 2023.

5 Interview (GKHZ) with Sibylle Köhler in Kranenburg, Germany, on September 10, 2022; BayHStA MK 43414.

6 The *Deutschnationale Volkspartei* (DNVP; German National People’s Party) was the major national-conservative party during the Weimar Republic before the rise of the *Nationalsozialistische Deutsche Arbeiterpartei* (NSDAP; National Socialist Workers’ Party). The DNVP rejected the republican Weimar Constitution of 1919 and the Treaty of Versailles and aimed at restoration of monarchy. After 1929, the DNVP co-operated with the NSDAP and supported Adolf Hitler’s appointment as *Reichskanzler* (Chancellor) in January 1933. In June 1933, the DNVP dissolved itself, with the majority of its former members joining the NSDAP. Several former DNVP members were involved in the plot to overthrow Hitler on July 20, 1944.

7 Notes written by Ruth Beutler in Munich around 1946 (provided by Sibylle Köhler).

8 Heinrich Himmler (1900–1945) was a leading politician during the Nazi era. The positions he held included *Reichsführer-SS*, Minister of Interior, and Chief of the German Police. He was also one of the main architects of the Holocaust.

9 BayHStA MK 43414.

10 UAM E-II-884. Ruth Beutler’s niece, Sibylle Köhler, remembers from conversations within the family that her aunt was initially wrongly treated for arthritis (interview with GKHZ in Kranenburg, Germany, on September 10, 2022).

11 UAM VA A-I-40-2.

12 In many universities in Europe and other countries, the chief executive is referred to as rector. The position is comparable to those of president at U.S.-American universities and of vice-chancellor at universities of Commonwealth nations.

13 Karl von Frisch used the Nazi ‘Heil Hitler!’ salute regularly in his correspondence with superiors at the University or authorities of the German Reich during the Nazi era.

14 BayHStA MK 43414.

15 Ibid.

16 Resume of Ruth Beutler; BayHStA MK 43414.

17 Ibid.

18 Richard Wilhelm Karl Theodor Ritter von Hertwig (1850–1937) was a German embryologist.

19 Short form for professor ordinarius. Until the 1970s, highest-ranked professorial position in Germany. As holder of a chair in a subject area (e.g., zoology), the *Ordinarius* defined the teaching of this area and was line-manager of lower-ranked professors affiliated with him.

20 Resume of Ruth Beutler; BayHStA MK 43414.

21 Ibid.

22 The first woman was Adele Hartmann (1881–1937), who had received her *Habilitation* in medicine in 1918. Hartmann was not only the first woman who received this highest academic degree from the University of Munich but also the first woman from any German university.

23 Resume of Ruth Beutler; BayHStA MK 43414.

24 http://www.documentarchiv.de/ns/beamtenges.html.

25 BayHStA MK 43414.

26 Ibid.

27 UAM E-II-884. After 1935, the autonomy of universities was severely restricted and professorial appointments required the approval of the Ministry of Science, Education, and National Culture in Berlin. This policy was part of the measures taken by the government under Adolf Hitler to ensure that professors were appointed not just based on scientific merit but also on their conformity with Nazi ideology. A central role in the latter process played *Dozentenschaften* (cf. Mertens L (2004) „Nur politisch Würdige “: Die DFG-Forschungsförderung im Dritten Reich 1933–1937. Akademie Verlag, Berlin). Initially founded by young Nazis as a representation of non-professorial academic staff at universities, later they included professors and assumed a far-reaching political mandate. By evaluating the ideological suitability of candidates and making recommendations, they played a critical role in the appointment of professors.

28 Wilhelm Führer (1904–1974), a German astronomer, was a leading figure in the *Dozentenschaft* at the University of Munich and the *Nationalsozialistischer Deutscher Dozentenbund*. In these functions, and as a bureaucrat in the Bavarian State Ministry for Education and Culture in Munich, and the Ministry of Science, Education, and National Culture in Berlin, he frequently pursued the dismissal of Jews and politically undesirable persons from their university positions. In parallel, he progressed through the ranks of the Waffen-SS, and served, among others, as aide-de-camp of Heinrich Himmler (https://ns-reichsministerien.de/2020/03/10/wilhelm-fuehrer/).

29 BayHStA MK 43414.

30 In his letter, Karl von Frisch used the word *Mitarbeiterin*, which indicates in German a female staff member. This wording leaves it open to interpretation whether he meant that Ruth Beutler was the most successful among his female staff members, or whether she was the most successful among all staff members, including men and women.

31 BayHStA MK 43414.

32 BayHSta MK 54482.

33 BayHSta MK 43414.

34 The *Nationalsozialistischer Deutscher Dozentenbund* (National Socialist German University Lecturers League) was established as a suborganization of the NSDAP in 1935. Its *Gau* (district) leaders played an important role in the appointment of faculty at universities, and in the dismissal of Jewish scientists or scientists whose political views did not align with Nazi ideology.

35 BAB R 4901/24579.

36 Ibid.

37 Ernst Bergdolt (1902–1948) was a German botanist and non-tenure track professor at the University of Munich.

38 BAB R 4901/24579.

39 In Nazi terminology, a Mischling of the second degree, or quarter jew, was (regardless of religious affiliation or self-identification), a person with one Jewish grandparent.

40 BayHSta MK 54482.

41 BAB R4901/24579.

42 *Das Reich* was a weekly newspaper founded by Joseph Goebbels, the propaganda minister of the Nazi government, in 1940. Its circulation grew to over 1.4 million by 1944, making it one of the most-read newspapers during the Nazi era. Goebbels himself contributed a weekly editorial. Unlike other Nazi newspapers, such as *Der Völkische Beobachter*, *Das Reich* was targeted at a more educated readership. The high journalistic quality of many of its articles contributed significantly to the intended glossing over the negative reality of the Nazi regime.

43 Based on pseudoscientific ideas about genetics, the Nazis proclaimed the need for *Rassenhygiene* (‘racial hygiene’, eugenics), involving the promotion of selective breeding of *Höherwertige* (superior people; under Nazi, ideology people of Nordic or Aryan traits), while excluding *Minderwertige* (inferior people; under Nazi ideology, for example, people with congenital cognitive and physical disabilities, and homosexuals) from reproduction. As a result, an estimated 400,000 people were sterilized against their will. Up to 300,000 people were murdered just in the *Aktion T4*, one of the major euthanasia programs.

44 Hans Spemann (1869–1941) was a German developmental biologist and *Ordinarius* for Zoology at the University of Freiburg from 1919 to 1937. Together with his PhD student Hilde Mangold, he discovered embryonic induction, a process through which a group of cells (the so-called ‘organizer’) directs the development of other cells. For this work, he received a Nobel Prize for Physiology or Medicine in 1935.

45 Bernhard Rust (1883–1945) was Reich Minister of Science, Education, and Culture from 1934–1945. He was instrumental in purging German universities of Jews and political adversaries. He also initiated a reform of Germany’s education system, aimed at immersing students into Nazi ideology.

46 BAB R 4901/24579.

47 Wilhelm Frick (1877–1946) served continuously in Adolf Hitler’s cabinet, first as Reich Minister of the Interior, then as Reich Minister without portfolio. He was instrumental in formulating several racist and antisemitic laws, including the Law for the Restoration of the Professional Civil Service in 1933 and the Nuremberg Laws in 1935. Frick was convicted of war crimes at the Nuremberg trial and executed.

48 Martin Bormann (1900–1945) was Chief of the Nazi Party Chancellery (the head office of the Nazi party) and Private Secretary of the *Führer*. In these two capacities, he became one of the most powerful Nazi officials during the Third Reich.

49 Ernst Bergdolt in a letter to the Reich Ministry of Science, Education, and National Culture, dated May 20, 1941; BAB R 4901/24579.

50 Letter of Martin Bormann to Bernhard Rust, dated July 11, 1941; BAB R4901/24579.

51 BAB R 4901/24579.

52 Infection of *Apis mellifera* by a second microsporidium parasite, *Nosema ceranae*, was first identified in 2004.

53 Franz Wirz (1889–1969) was a German dermatologist and Nazi official who served in various leadership positions, including in the *Hauptamt für Volksgesundheit der NSDAP-Reichsleitung* (Head Office for People’s Health at the NSDAP Headquarter. After World War II, Karl von Frisch intervened on behalf of Wirz. In a letter dated, January 10, 1946, and addressed to the commanding officer of the Counter Intelligence Corps (a World War II and early Cold War intelligence agency within the United States Army) Detachment in Munich, he wrote: *I have received news that Professor F. Wirz was apprehended end of December. I do not know what charges were brought against him. However, I would like to share some information with you in his defense. When, in 1940, the Reich Minister of [Science,] Education [, and Culture] had the intention to force me to retire, due to a non-Aryan grandmother, many people made great efforts so that I could remain in office, yet without success. When Professor Wirtz learned about this issue, he intervened vehemently on my behalf. It was him who made arrangements, so that my forced retirement was delayed until the end of the war… At his request, the Reich Minister of Food and Agriculture argued that, because of the importance of my research on bees for nutrition, they cannot afford losing me. Of course, Professor Wirz knew that I was not a member of the [Nazi] party, and that I was even opposed to its ideology. Nevertheless, he strongly supported me…* (BSB, Ana 540.C.III.23.).

54 Richard Walther Darré (1895–1953) was Reich Minister of Food and Agriculture from 1933 to 1942.

55 BAB R 4901/24579.

56 Franz Wirz summarizes some of his interventions on behalf of Karl von Frisch in a letter to Ministerialrat Küthe of the Reich Ministry of Food and Agriculture, dated December 17, 1941; BAB R 4901/24579.

57 Letter of Bernhard Rust to Richard Walther Darré, dated February 10, 1942; BAB R4901/24579.

58 BAB R 4901/24579.

59 Letter of Martin Bormann to the Reich Minister for Science and Education, dated April 27, 1942; response of the Reich Minister of Science and Education to the Head of the Party Chancellery, dated July 27, 1842; BAB R4901/24579.

60 Karl von Frisch is quite vague in his description of the approach he had proposed. What he appears to have suggested to Ruth Beutler is that, instead of first training scout bees to petri dishes associated with a particular scent near the hive and then moving these dishes gradually to more distant locations, she should simply train the bees to a scented feeding place near the hive, and then instantly move this setup to the distant location. Since, according to his hypothesis postulated in 1923, the dances of the bees do not contain information about the distance of food sources from the colony, he expected hive mates alerted by the scout bees to fly out to search for the odor associated with the new food source until they had arrived at the distant spot.

61 Increasing evidence over the last few decades has questioned the dichotomy of the round dance versus the waggle dance, as postulated by Karl von Frisch in both his original model of 1923 and the revised model of 1946. Such evidence includes the presence of waggle phases, as well as information about distance and direction, in round dances (Kirchner WH, Lindauer M, Michelsen A (1988) Honeybee dance communication: acoustical indication of direction in round dances. Naturwissenschaften 75:629–630; Jensen IL, Michelsen A, Lindauer M (1997) On the directional indications in the round dances of honeybees. Naturwissenschaften 84:452–454; Gardner KE, Seeley TD, Calderone NW (2008) Do honeybees have two discrete dances to advertise food sources? Anim Behav 75:1291–1300). These observations have led to the hypothesis that round dance and waggle dance are just the ends of a continuum, with the information communicated by the bees becoming less precise the closer the food source is to the hive (Gardner KE, Seeley TD, Calderone NW (2008) Do honeybees have two discrete dances to advertise food sources? Anim Behav 75:1291–1300). Moreover, even when food sources are at distant locations from the hive, the information conveyed by the returning bee indicates only the approximate location of the food. Additional pieces of information, such as the scent of the flowers that the dancing bee carried to the hive, and the pheromones of the Nasonov gland that this bee released when it discovered the new food source, are necessary for the recruited bee to find this source of food (for review see Tautz J (2022) Communication between honeybees: more than just a dance in the dark. Springer Nature, Cham). Interestingly, already Karl von Frisch described wagging during the round dance (p. 12, von Frisch K (1946) Die Tänze der Bienen. Österr Zool Zeitschr 1:1–48), but he did not attribute any functional significance to this behavior.

62 Albert Maucher (1907–1981) became *Ordinarius* and Director of the Geological Institute at the University of Munich in 1947, after he had served as Interim Director for one year.

63 Siegfried Walter Souci (1904–1992) was Interim Director of the Institute of Pharmacy at the University of Munich from 1946 to 1947.

64 Karl Suessenguth (1893–1955) was Interim Director of the Botanical Institute at the University of Munich from 1945 to 1948.

65 In her letters to Karl von Frisch, Ruth Beutler frequently used abbreviations of last names. Here, she made reference to Klaus Clusius (1903–1963), Dean of the Faculty of Science. He was *Ordinarius* and Director of the Physical Chemistry Institute at the University of Munich from 1936–1947.

66 Letter of Ruth Beutler to Karl von Frisch, dated July 2, 1947; BSB Ana 540.B.IV (Beutler, Ruth).

67 BayHStA MK 43414.

68 Ibid.

69 Fritz Machatschek (1876–1957) was an Austrian geographer and *Ordinarius* at the University of Munich from 1935 to 1946.

70 Paul Buchner (1886–1978) was a German zoologist and cell biologist, and a friend of Karl von Frisch. He received his PhD and *Habilitation* from the University of Munich. His research focus was on endosymbiosis.

71 Otto Koehler (1889–1974) was a German zoologist and ethologist, and a friend of Karl von Frisch. He received his PhD from the University of Munich. His research interests included a wide range of biological phenomena in a variety of organisms. He became best known for his demonstration of a pre-linguistic ‘number sense’ in several animal species, particularly birds.

72 Johann Heinrich Wilhelm Goetsch (1887–1960) was a German zoologist whose research focus was on social insects.

73 BayHStA MK 69782.

74 Ibid.

75 Ibid.

76 Ibid.

77 After the unconditional surrender of the Nazi German armed forces to the Allies, Germany was divided into four military occupation zones — the American, British, and French zones in the West, and the Soviet zone in the East. Similarly, Austria was split into four zones. Allied Military Travel Permits were required for travel between the zones and across borders.

78 Letter of Hans Rheinfelder to Karl von Frisch, dated July 16, 1948; BayHStA MK 69782.

79 BayHStA MK 69782.

80 Ibid.

81 Erich von Holst (1908–1962) was a German behavioral physiologist and founding director of the Max Planck Institute for Behavioral Physiology in Seewiesen.

82 BayHStA MK 69782.

83 Ibid.

84 Ibid.

85 Otto Jessen (1891–1951) was a German geographer and *Ordinarius* at the University of Munich.

86 BayHStA MK 69782.

87 Letter of Karl von Frisch to the Bavarian State Ministry for Education and Culture, dated February 6, 1950; BayHStA MK 69782.

88 Hansjochem Otto Autrum (1907–2003) was a German animal physiologist. He succeeded Karl von Frisch as *Ordinarius* at the Zoological Institute of the University of Munich. He was Editor-in-Chief of the Journal of Comparative Physiology A from 1960 to 1996.

89 Heinrich Otto Wieland (1877–1957), *Ordinarius* for Organic Chemistry at the University of Munich from 1925 to 1952. He received a Nobel Prize for Chemistry in 1927. Wieland is known for his public opposition to the Nazi regime, as well as his protection of Jewish scientists and members of the White Rose resistance group in his lab.

90 UAM OC-VIII-5 Bd. 8.

91 Letter of Ruth Beutler to Karl von Frisch, dated July 6, 1947; BSB Ana 540.B.IV (Beutler, Ruth).

92 Letter of Hans Meinzolt to Theodor Kollmann, dated March 29, 1950; BayHStA MK 69782.

93 Der Spiegel, February 20, 1948, https://www.spiegel.de/politik/professor-dr-heinrich-wieland-a-c764a8ed-0002-0001-0000-000044415717, retrieved September 8, 2022.

94 In a letter to the Bavarian State Ministry of Education and Culture, dated December 12, 1949, Karl von Frisch had listed his conditions for accepting the offer of appointment. His first condition was the *Konservator* position for Ruth Beutler. Although not all of his other conditions were met by the State Ministry, he accepted the offer and returned to Munich in 1950. However, in a letter dated June 26, 1951, he informed the State Ministry that he had received an (informal) invitation to join the University of Vienna as *Ordinarius* for Zoology. He repeated two previously presented requests as a condition for not further considering a possible offer from the University of Vienna, and he demanded an immediate answer; UAM E-II 1376.

95 Letter of the Bavarian State Ministry of Education and Culture to the rector’s office of the University of Munich, dated December 14, 1950; UAM PA allg. 26.

96 https://stadtgeschichte-muenchen.de/strassen/d_strasse.php?id=6063

97 BSB Ana 540.B.II (Beutler, Ruth).

98 BSB Ana 540.B.IV (Beutler, Ruth).

99 A letter of recommendation (BSB Ana 540.C.III.72), written by Karl von Frisch on behalf of Ruth Beutler, adds some information related to this question. The letter was, most likely, written on June 20, 1947 (on the carbon copy of the typed letter, only one digit of the year is visible, ‘7;’ considering the context of the letter, the year appears to be ‘1947’) and addressed to Alfred Kühn. Karl von Frisch acknowledges the work of Beutler, in her function as his deputy, …*which is almost too much for her to bear*. He, then, raises the question of what her career prospects will be after this interim position. He writes that …*based on her accomplishments, she deserves, without any doubt, to be appointed to an Ordinarius position*. On the other hand, his letter also indicates that his support is not unrestricted. *In times like this, the pool of suitable candidates is very small. It would, therefore, be quite justified to entrust a woman with fulfilling the responsibility of an Ordinarius. I believe she [Ruth Beutler] would be up to the job if it were a smaller university, such as Erlangen.* In concluding his letter, Karl von Frisch asks Alfred Kühn for his support in this matter, if he consents with the assessment of Ruth Beutler’s qualification.

100 Margarete von Wrangell (1877–1932) was the first female *Ordinarius* in Germany. She received her PhD in organic chemistry from the University of Tübingen in 1909 and was appointed to an *Ordinarius* position in plant nutrition at the *Landwirtschaftliche Hochschule Hohenheim* (today known as the University of Hohenheim) in Stuttgart in 1923.

101 Nagler-Springmann (1997) cites in her essay the original German version of the book (Deichmann U (1992) Biologen unter Hitler. Campus Verlag, Frankfurt/Main). The quote here is taken from the English translation of her book (Deichmann U (1996) Biologists under Hitler. Harvard University Press, Cambridge).
